# Book Reviews

**Published:** 1808-04

**Authors:** 


### CRITICAL ANALYSIS

### OF THE

### RECENT PUBLICATIONS

### ON THE

### DIFFERENT BRANCHES OF PHYSIC, SURGERY, AND MEDICAL PHILOSOPHY.

---

*An Inquiry into the Changes induced on Atmospheric Air, by the Germination of Seeds, the Vegetation of Plants, and the Respiration of Animals.* By DANIEL ELLIS. 8vo. Edinburgh.

WE have been so much accustomed to what is called *reasoning*, even in the cool temperature, to which we owe the present production, that we seized with peculiar satisfaction a detail of operose experiments and fair inductions. The author informs us, that he was led to these investigations by observing the spontaneous recovery of an animal, in whom all the appearances of life had been suspended by drowning. Instead of satisfying himself with the theories which have been proposed to explain such a phenomenon, he determined to consider with particular attention the physiology of respiration. In the course of his enquiry, analogy suggested a comparison of the facts ascertained in human respiration with those which have been observed in inferior animals, and hence to changes produced by the support of vegetable life. Thus he observes, in a descending series, all the great classess of animated nature were successively brought under review; "and arriving at the most simple form of existence, he was led to make it the first subject of investigation, and then to retrace his steps through the more complex and perfect forms of vegetable and animal matter." We may add, that after a very careful perusal, we recollect no instance in which he has formed conclusions, however plausible, without confirming them by every experiment, supported by every authority, and meeting every objection that the nature of the subject seemed to require.

The First Chapter is of the changes induced on the air by the germination of seeds. The experiments are so numerous, and conducted with such apparent accuracy, as to leave no room to doubt the result as stated by our author. But as, on all these occasions, it must be the wish of a just experimenter that the reader should draw his own inferences, we shall take a liberty which implies a compliment, inasmuch as it admits the faithfulness of every report.

And first, we must give Mr. Ellis full credit for proving most satisfactorily, that the same effects are produced on the atmosphere by the germination of seeds, the vegetation of plants, and the respiration of animals. These facts are highly important, and serve to show how much better founded is what was once thought a vulgar error, than the supposed improvements of some philosophers. That the air emitted by plants is injurious, has been a long received notion. The founder of the pneumatic chemistry, always faithful in his narrations, but often hasty in his conclusions, conceived he had at once discovered the source by which the atmosphere was purified after the respiration of animals. In this he was more generally followed than in most of his opinions. It is not to be wondered if he died in his errors, as he was never satisfied with his own discovery concerning the composition of water. But it has been long suspected that the improvement in the air arose from the decomposition of water, and not from the process of vegetation; and Mr. Ellis's experiments show that the vulgar opinion is well founded, and that the air is actually deteriorated by every part of the process of vegetation.

But another and very important difficulty remains. What becomes of the oxygen, without which, none of the above living processes can be maintained, and which disappears during the progress of such processes? Mr. Ellis is at great pains to show that this cannot be by the absorption of oxygen; and also, that carbonic acid cannot be excreted: he therefore concludes, that carbon is excreted in minute portions, which, combining with the external oxygen, either pure, or as a part of the common atmosphere, produces carbonic acid. Our readers may be startled at so bold a theory, and a vast number of difficulties will at once occur to them. It would be great injustice not to remark, that Mr. Ellis has, by a vast variety of experiments, and by every authority he could collect, met, we believe, every objection that can be started; but to show this in his own way, would be to offer extracts too unconnected to be interesting, or too copious for our limits. It is enough to say, that all who feel sufficiently interested in the subject will peruse the work itself. We shall select the following as a passage more easily disjointed, yet containing the general result of the arguments on the aerial changes induced by living animals.

"Ascending next, in the order of our inquiry, into the animal kingdom, we find, that, to insects and worms, the presence of oxygen gas is essential to the continuance of living action (53. et seq.) and that they gradually, and, in many instances, completely convert this gas into carbonic acid. No other substance but the animal being present in the experiment by which the carbon could be supplied, we must refer its production to that source. The formation of this acid was found also by Spallanzani to depend very much on temperature; for snails consumed more oxygen, and died in equal bulks of air, much sooner when the temperature was mild, than when it was severe; and, by gradually reducing the temperature, he at length reached that point where the air in which the snails were confined, underwent no change at all; which point he found to be at zero. The circulation all this time was gradually declining, and at —1° Reaum. the pulsations altogether ceased, the the heart remained perfectly at rest, and the whitish fluid usually flowing in the vessels was in a state of stagnation. Nearly similar results were obtained from another species of snails in a hybernating state. In these, the covering or opercle with which they close up their shell in winter, effectually excludes the air on that side, neither does it penetrate their shells elsewhere; for a small glass tube, filled with mercury, being inserted into this covering, and then inverted, sustained a column of 28 inches of that fluid, which corresponded to the height of the mercury in an adjacent barometer. When, however, the covering was punctured so as to admit the air, it instantly forced down the mercury. The small portion of air contained in these shells for several months during the torpid state of the animals, was found in several trials to be of like purity with that of the atmosphere; so that when living action had ceased in these animals, no change was produced on the air in contact with them.

"Nor are these effects peculiar to those animals which change the air by the mediation of their skin: they are common also to those which breathe by lungs. A marmot that was cold to the touch, and so closely rolled up as to be tossed about and irritated in every shape without exhibiting the slightest indication of life, was exposed in a recipient of atmospheric air, inverted over mercury, to a temperature —12° Reaumur. He continued motionless three hours and a half, during which time the mercury in the jar remained stationary: and the air, on being analysed, was found in every respect similar to that of the room in which the experiment was made. The same animal was then transferred into a jar of carbonic acid at temperature —12.5° Reaumur, where he remained four hours without shewing the least sign of motion, or suffering the smallest injury; but a rat, put at the same time into the jar, died almost instantly; and when, in a second experiment, the temperature was raised to zero, the marmost almost began to breathe, and then speedily died in a jar of this gas. These facts prove, not only that the carbon, which decomposes the air, is in every case furnished by the animal, but that its emission depends wholly on the state of the circulating fluids; for, as the circulation increased, declined, or ceased, so likewise did the emission of carbon, and consequent production of carbonic acid: the oxygen gas could not vary the result, being in all cases equally abundant.

"Does then this emission of carbon proceed from the fluids circulating in the larger vessels of the animal? Or is it given out from the exhalent structure of the body? That these fluids may contain carbon as a constituent element, we do not mean to deny: but, that this substance can be emitted through that organized structure which carries on the respiratory function, may, we think, be justly disputed. This structure has been shewn to embrace a large portion of the surface of the bodies of insects, which again is occupied only by the terminations of exhalent vessels, whose office it is to emit fluids, previously separated from the blood, while the blood itself usually circulates in the deeper seated vessels, or even at the centre of the animal. When also the usual changes on the air are suspended, by placing the animal in a cold medium, the carbon might be expected to accumulate in the blood, if that were the source from which it is directly derived, and carbonic acid to be at that time most abundantly produced: but the preceding facts evince, that it is not to the supposed accumulation of this substance in the blood, but to the state and vigour of the circulating fluids that the production of this acid is to be ascribed. We have therefore no proof that carbon exists in the mass of blood in a state capable of attracting air, or of being attracted by it through the organized structure of the animal: nor even if these attractions were allowed to take place, do the phenomena at all correspond with what, under such a supposition, ought to happen.

"If, then, the carbon in question be not emitted from the blood while yet in the larger vessels, nor after it has ceased to be in motion, it must be given out by that fluid while in circulation, and after it has entered into the minuter vessels: and thus it becomes an animal excretion, derived, like other excretions, from the blood, and emitted, like them, by some appropriate structure from the surface of the body. Hence any cause, as cold, checking the circulation, restrains the production of this carbon; or, although the circulation be not checked, the emission of this substance is prevented by smearing the bodies of insects over with unctuous matter, which in consequence causes their death. All that has now been said of insects and snails, applies equally to the marmot and other torpid animals; for the emission of carbon in all, is obedient to the same laws, except that, in the former case, it is given out by the exhalent surface of the body, while, in the latter, it proceeds from the exhalent structure of the lungs. For these reasons, we consider the emission of carbon in these animals to be truly an excretion dependent on the due circulation of their blood, and partaking of all its variations. The experiments of Spallanzani, it may be observed, prove likewise, that under decomposition, animal as well as vegetable substances form carbonic acid in air where no oxygen gas was originally present; a fact with which the experiments of Priestley had long before made us acquainted."

From these and many other experiments and authorities, our author concludes, that the excretion of carbon is an animal function dependant on life, but which the animal is incapable of performing without the presence of oxygen, either pure or diffused, in some other air. He at the same time conceives, that the living membranes are incapable of transmitting oxygen, and also not only that no means of absorption of oxygen have yet been discovered, but that we have not sufficient proof that it has ever been either absorbed or transmitted in its pure state by any part of the living body.

By our author's account, it would appear that the principle, if not not the only use of oxygen in transpiration and respiration is to enable the animal to discharge excreted carbon, which he supposes both by the skin and the lungs is emitted with water. The transpiration of carbonic acid, as proved by Mr. Abernethy, he conceives a fallacious experiment, and shows that Drs. Klap and Woodhouse repeated it with a result completely different.—That the black colour in the blood is the effect of carbonic acid, is shown to be destitute of proof.

"If, then, (says our author) there be no proof that oxygen gas enters into the body through the skin, and if it be also allowed that no carbonic acid can be emitted from that organ, it must be granted, that the acid actually produced, is formed exterior to the skin itself; and the disappearance of the oxygen gas, in proportion to the production of this acid, leads at once to the opinion that it is formed in part out of that gas. M. Jurine found accordingly, that as much acid was formed out of a given bulk of air, when his arm was confined in it for one hour, as when the experiment was continued for three or four hours, which, it appears to us, could have arisen only from all the oxygen gas having within the first hour been changed. For the formation of this acid, however, carbon, its other ingredient, must be in some way supplied; and as, in these experiments, no other substance but the skin was in contact with the air, from which it could be derived, we must ascribe the production of the acid to the spontaneous union of carbon, furnished by the skin, with the oxygen gas of the air; and this carbon we conceive to be given out by its exhalent function.

"In vegetables, and in the inferior animals, it has been shown, that carbonic matter is yielded only while the circulation of their fluids continues; and there are some facts related by M. Jurine, which render it very probable that the same thing holds true concerning the carbon which is furnished by the human skin. He found, that in given times, the greatest quantity of carbonic acid was formed by the skin of a person in the vigour of life; next by a boy, ten years of age; and least of all by a man of 70: and that its production depended much, likewise, on the season of the year, and the vigour of the cutaneous organs. All these facts seem necessarily to follow from the opinion that this carbonic matter is truly an excretion, depending altogether on the distribution of the animal fluids, and subject to all their variations. Whether, as in other animals, the air is no longer decomposed by the skin, when the circulation has wholly ceased, we have not the means of deciding; but the results of the foregoing experiments of Jurine, lead us to suppose that such is actually the fact. Even at the period in which Mr. Cruickshank wrote, it is probable that, had the composition of carbonic acid been then determined, he would have formed a similar opinion concerning the source of the carbon: for, in referring to his experiments, he observes, 'that (admitting the common theory of fixed air and phlogiston) some-

 thing thing passed off with the vapour of insensible perspiration by the skin, which rendered air fixed, and heavier than atmospheric air.' By wearing a fleecy hosiery vest also next the skin for a month at a time in the hottest parts of summer, he collected an oily substance, which accumulated on the nap: this substance he exposed to a red heat in a silver spoon, and, when burnt, it left behind a black powder, resembling in every thing the powder of charcoal. 'This all seems to prove,' he adds, 'that phlogiston' (by which he can here mean nothing but carbon) 'is emitted from the pores of the skin.' From the establishment of this power in the skin, we obtain, besides, this additional and important fact,—that carbonic acid may be formed out of the oxygen gas of the atmosphere, when brought into contact with the human body, without the necessity, or even the possibility, of believing that the carbon entering into its composition, is derived from the venal blood; for the exhalent vessels of the skin are all of arterial structure, and the fluids which they contain and emit, exhibit nothing of the colour or the properties of venal blood."

Whilst we admit, with our author, the difficulties of proving that oxygen enters the human body either by absorption or by its affinity with blood, candour obliges us to acknowledge, that we cannot completely make up our mind as to the cause of that necessity which every animal or vegetable feels for oxygen. We consider Mr. Ellis's experiments as conclusive, like many others, in the negative he wishes to establish; but that much more is wanted to ascertain the nature of these processes, by which the lungs or skin are found to produce their well known changes in certain elastic fluids. We allow the difficulty of proving absorption; and as to mere chemical attraction through a living membrane, however thin, we are forced to admit, that if such a power of transmission does exist, it does not increase with health and strength, as Mr. Ellis has shown to be the case with that process by which carbonic acid is formed. We are told, that the jugular vein of a dog was laid bare, the surrounding cellular membrane removed, a stream of oxygen blown on the vein, and notwithstanding the small surface of blood to which the oxygen was applied, that the colour of the blood was considerably heightened. We trust the experimenter employs his talents better than in so uncertain an operation. Let him compress the veins of a dog till the tongue becomes black, and see whether by exposure to common air, or by a stream of oxygen, the tongue will recover its colour. Let him view the countenance of a man previously healthy, under suffocation from choking, or any other cause; does the lip recover its colour from the surounding atmosphere? Let him see the same man after death; and if his friends are by, let him hear them express their astonishment at the recovery of their friend's complexion.

Whilst we offer these arguments in favour of our Author's inductions, and in answer to an experiment, of the nature of which we are are by no means master, we can only encourage Mr. Ellis to pursue his enquiries with the same diligence and ingenuity as appear in every part of his volume. If we are not always satisfied with his inductions, we confess we have nothing better to offer, and that he has convinced us of the errors of many of his predecessors, as well as of our own.

---

*A Popular View of Vaccine Inoculation, with the Practical Mode of conducting it. Shewing the Analogy between the Small-pox and Cow-Pox, and the Advantages of the latter. By* Joseph Adams, M. D. F. L. S. Physician to the Small-pox and Inoculation Hospitals, and to the Finsbury or Central Dispensary. 12mo. London. 1807.

It is long since we have met with the subject of Vaccination treated with the true temper of philosophic enquiry. Invective has supplied the place of reasoning; and accusations of murder have been retorted on each side, with an indelicacy rather to be expected from Billingsgate, than the votaries of science, or the practitioners of an useful art. From the present work, as the production of the author of Morbid Poisons, and physician to that hospital which first reconciled the public to vaccination, we might expect, as the title professes, every practical lesson. We might also expect a philosophical review of the causes to which we may attribute the manner in which one disease is thus made, if not vicarious to, at least a security against, another. In our review of this popular treatise, we shall principally attend to those particulars which are not contained in former publications on this hacknied subject.

The first chapter contains introductory and historical observations on the probable period at which small-pox first appeared in Europe; and the consequences of it not only on the health, but opinions of mankind. Among the arguments for supposing that small-pox was unknown to the ancients, we shall extract the following, as we believe altogether new.

" It is not less certain that such of the ancients as describe infectious fevers, were totally ignorant of one property which could not have escaped them, had the small-pox been then known; namely, that no person is liable to the disease more than once during life. Thucydides, in his justly celebrated account of the pestilence which ravaged Athens with uncontrouled fury, informs us, that few were attacked a second time, and none with severity: and that this appeared a matter of such astonishment, that the convalescents fancied themselves for ever after exempt from all diseases. Now it is well known, that though the constitution is not secured from a second attack of the plague, yet that such a recurrence during the same season, is very uncommon. This is so far from surprising the Europeans in these days, that most of us express our astonishment, that the disease should occur a second cond time to the same person, at a period however remote. It seems to me impossible to account for the different impression excited by the same event in any other way than by that change in our mode of viewing infectious diseases, which our acquaintance with the small-pox has introduced amongst us.

"It is highly probable that the first introduction of this contagion was by the crusades. Ambrose Parey assures us, that after that event, the leprosy became so frequent, that not less than 21,000 lazarettos were erected in different parts of France. It is difficult to conceive that these could be for any other purpose than for the reception of those afflicted with so general and so contagious a distemper as small-pox. This is the more probable, because as it was soon found, that in populous towns it was scarcely possible to avoid the disease, and that such as had once passed through it, were no longer in danger, many of these lazarettos gradually fell into disuse, till at length the introduction of inoculation rendered them no longer objects of general attention."

The author next enters into the enquiry, whether the introduction of small-pox inoculation has lessened the numbers who have died under that disease. This he concludes, by observing in general, that it has rendered the lives of most prudent people more comfortable, by securing themselves and friends from the terrors of such a malady; and that the Jennerian discovery has rendered the question of comparatively less consequence. He proceeds next to trace the first introduction of inoculation; the objections made to it; the means by which those objections were obviated, and the practice at last generally adopted, in consequence of the improvements of the Suttons, the Dimsdales, and others.

In the next chapter Dr. A. undertakes to show the causes to which we may attribute the advantages of receiving small-pox by inoculation, rather than in the casual way. These he attributes to the first shock of the disease being confined to a small spot in the arm, rather than diffused over the whole face and neck, as in the casual disease. That the whole danger depends on the first shock, he conceives, is proved by the observation of Sydenham, since confirmed by all subsequent writers, namely, that nothing is to be apprehended excepting from the number and complexion of the pustules in the face, and from the period at which they appear: by which we may form a certain prognosis of the future progress of the disease. Another argument in favour of this opinion, is the progress of the disease under inoculation, in which it is observed, that if the eruption on the face should not appear later than at the part of insertion, the patient receives no material benefit from the operation. He conceives it probable, too, that the Suttons were, at the beginning of their practice, indebted to chance for other advantages by which the popular opinion became so universally in favour of them and the practice of inoculation.

This advantage, it afterwards appears, the author imputes to the insertion of a favourable kind of small-pox. Besides the grand grand distinction of Sydenham between the confluent and distinct, there are, it seems, others which might be made; and that some of these are permanent in producing a similar disease by inoculation. The most favourable of all, we are told, approaches the nearest in its appearance and laws to vaccination; and that it is not easy to distinguish the best small-pox from the true vaccine vesicle. A presumptive proof that the cow-pox and small-pox are the same poison, is drawn from those laws which the author has detected in other morbid poisons. By these it appears, that the chicken-pox and measles will interrupt the progress of each other, or of small-pox or cow-pox: yet, that the two latter will proceed together without interruption, subject to exactly the same laws as if only one were inserted.—Having thus completed the analogy between the two diseases, and the probable causes of the advantages derived from inoculation, the author proceeds to the practical part of the work, or the mode of conducting small-pox inoculation. This he divides into—The season of the year; the state of the person to be inoculated; the choice of the variolous fluid; the manner of inserting it; and lastly, the treatment of the patient. For the season of the year, contrary to general opinion, Dr. A. prefers Summer, remarking, that the Register of the Hospital proves that the disease, both in the casual and inoculated way, is invariably milder at that season. This may seem to contradict the well-established fact, that the application of cold is serviceable in small-pox; but this is explained, by showing that it is only in warm weather that successive changes of air can be applied to the human body. In cold weather it will be almost impossible to prevent the patient, and even the nurse, from preserving the air heated by the body and impregnated with the variolous poison. Whatever may be the cause, we cannot dispute the fact on such authority. As to preparation, the author objects greatly to a reduced state of health, and thinks it sufficient to begin with a few powerful purges on the morning after insertion, repeating them as the constitution or other circumstances may require. The matter, he advises, should always be fresh, when such can be procured, taken at an early period of the eruption, before it is become purulent. Where there is a choice of the kind of small-pox, the pearl, or white sort, should always be preferred. The prognostic symptoms of the future disease are next enumerated much at large, and the mode of treatment under each.

Having thus gone through the subject of small-pox inoculation, to show the advantages of vaccination, it is thought enough to remark, that, almost all the cautions enumerated under the former practice are unnecessary in this. An enumeration of the objections against vaccination next follows. These have been so frequently discussed, that it is not to be expected the reader can meet with any thing very new. The same may be said of the succeeding Chapter, which contains a state of the question, Whether vaccination is a security against the small-pox? Another enquiry follows:

follows: "Are the marks of vaccination more uncertain than those of small-pox inoculation; and how are we to estimate the security of the latter?" This Chapter is perhaps the most important part of this division of the work. It is much too long to permit our transcribing it; and it may easily be conceived, will not admit of any abridgment.

The mode of treatment during vaccination, though for the most part of little consequence, it is remarked, should always be attended to: not only in order to ascertain the safety of our patient, but because it is well known that in some constitutions, inflammation excited by any cause is often alarming. The work concludes with a description of those deviations from the usual course of the disease, which are ascertained to be unimportant, such as render the operation doubtful, and such as are proofs of insecurity.

An Appendix is added, containing, first; the Report of the London College on vaccination. 2dly, The correspondence between the College and Dr. Adams. 3rdly, A description and history of that kind of small-pox which most resembles cow-pox. And, lastly, On secondary vaccine vesicles. The last being on a subject much disputed, we shall extract, in the author's words.

"As many persons who have considerably engaged in vaccination, profess never to have seen secondary vesicles, (vaccine eruptions on parts of the body remote from the first insertion) and as their appearance adds another proof of the analogy between the vaccine and variolous poisons, it may not be amiss to say a few words on the subject.

"There is no reason to doubt that the secondary eruptions remarked by Dr. Woodville at the beginning of the practice were variolous, and in many instances arose from the inoculation of small-pox matter a few days after vaccination; a very reasonable precaution, considering the seat of so new an experiment. The controversy produced on this occasion between that gentleman and Dr. Jenner ended in both parties admitting, that about the end of the second, or commencement of the third week, when the inoculated part is beginning to scab, a few scattered eruptions will appear, having all the properties of the original vesicle. By some it has been asserted, that these secondary vesicles arise from the fluid of the first being carried by the patient's finger to the place where they appear. But this can only be the case when such vesicles appear at an earlier stage, in which case they dry and scab precisely at the same time as the first insertion; whereas secondary vesicles do not appear till about the time when the first insertion is beginning to dry or scab. On this account they may sometimes occur without the knowledge of the vaccinator, who frequently sees nothing of his patient after the areola subsides and the scab begins to form itself in a satisfactory manner.

"A more striking resemblance between the two diseases, though a much more rare occurrence in cow-pox, is what may be called a crop of secondary eruptions. I do not recollect that these have been recorded by more than three writers. The first who noticed them was the Rev. Mr. Holt, whose patient had one hundred secondary pustules, from some of which he inoculated eight others and produced the genuine disease.—This is related in Med. Journal, vol. ii. p. 402. The second instance is to be met with in the same Journal, vol. ix. p. 309, being an account transmitted by the author of two cases of full vaccine secondary eruptions which occurred in the island of Madeira. The third is by M. Hallé, and to be met with in the Med. and Chirurgical Review, vol. xv. p. vi. Miscell. In this paper the author notices several anomala which appeared during a general vaccination at Lucca in Italy; among the rest he remarks, "eruptions of pustules over the whole surface of the body which took place at the time of the appearance of the areola around the inoculated part. These eruptions, which might easily be mistaken for variolous, differed however essentially from them in the manner of their formation, in the order in which they dried away, and especially in the nature of the fluid they contained, which was in no instance truly purulent. The dissimilarity was rendered a matter of demonstration by inoculation: for not only this fluid did not propagate any thing like small-pox, but from some observations there appeared reason to believe, that it was capable of producing the genuine vaccine vesicle."

Such are the contents of this little volume, which it can hardly be necessary to recommend to our readers. The importance of the subject is undisputed; and the opportunities of our Author are not less admitted.

